# Hepcidin Is a Reliable Marker of Iron Deficiency Anemia in Newly Diagnosed Patients with Inflammatory Bowel Disease

**DOI:** 10.1155/2020/8523205

**Published:** 2020-11-28

**Authors:** Milica Stojkovic Lalosevic, Ljubisa Toncev, Sanja Stankovic, Sanja Dragasevic, Stefan Stojkovic, Ivana Jovicic, Milos Stulic, Djordje Culafic, Tamara Milovanovic, Marija Stojanovic, Marko Aleksic, Mihailo Stjepanovic, Jovan Lalosevic, Stanimir Kiurski, Branislav Oluic, Aleksandra Pavlovic Markovic, Mirjana Stojkovic

**Affiliations:** ^1^Clinic of Gastroenterology and Hepatology, Clinical Center of Serbia, Belgrade, Serbia; ^2^Faculty of Medicine, University of Belgrade, Belgrade, Serbia; ^3^Center for Medical Biochemistry, Clinical Center of Serbia, Belgrade, Serbia

## Abstract

**Results:**

There was a high statistically significant difference between IBD patients and controls in levels of hepcidin (*P* < 0.01). Namely, serum hepcidin levels were significantly higher in the control group. There was no statistically significant correlation of serum hepcidin with CRP, Mayo score, or CDAI, respectively (*P* > 0.05). However, we have found a statistically significant negative correlation of sTfR and TIBC with hepcidin (*P* < 0.01).

**Conclusion:**

Results of our study suggest that hepcidin is a reliable marker of IDA in patients with IBD, and it could be used in routine clinical practice when determining adequate therapy in these patients.

## 1. Introduction

Anemia is one of the most common complications of inflammatory bowel disease (IBD), with the prevalence up to 70% at the time of diagnosis establishment [1, 2]. Taking into account its high prevalence, this common extraintestinal manifestation has a major impact on the quality of life in IBD patients. Therefore, an adequate and well-timed treatment is of great importance. Usually, treatment is determined by the etiology, and in the majority of the patients with IBD, anemia may be due to iron deficiency (IDA) or inflammation [1]. Differentiating these two most common etiologies represents a clinical challenge.

IDA is the most common anemia in general population, which is usually defined as a state of insufficient iron stores, subsequently affecting normal production of red blood cells. IDA in IBD patients frequently results from intestinal blood loss and reduced iron absorption from inflamed enterocytes [3]. Beside low serum iron levels and high total iron-binding capacity (TIBC), low serum ferritin and saturation of transferrin are the most commonly used blood parameters in the detection of IDA [4]. Anemia of chronic disease (ACD) is defined as a state of low iron availability, where the level of erythropoietin is moderately reduced and the lifespan of red blood cells is shortened. It is frequently associated with chronic inflammatory states such as diabetes mellitus, heart disease, rheumatoid arthritis, and other diseases with long-standing nature. ACD is usually normochromic, with normocytic or light microcytic erythrocytes, with low or normal serum iron, and low or normal total iron-binding capacity (TIBC) [5]. Additionally, ferritin levels are normal or increased with low reticulocyte count. Treatment of these two most frequent types of anemia also differs, in a manner where ACD does not respond to iron, folic acid, or B12 vitamin treatment, and the treatment of the underlying cause is necessary [6]. Patients with IBD represent a clinical challenge when it comes to differencing between IDA and ACD, since serum ferritin used for measuring body iron storage is an acute-phase reactant and is often elevated in systemic inflammation [7]. Moreover, IDA and ACD can overlap in the same patient, making the adequate diagnosis and timely treatment even more challenging.

Hepcidin is a peptide molecule, predominantly synthesized by hepatocytes, with a very important role in regulating processes of iron absorption and mobilization from the body stores. Hepcidin production is mostly regulated by the status of iron and inflammation [8]. An inflammation of any cause, especially IL-6 cytokine production, induces hepcidin synthesis. Hepcidin inhibits ferroportin, a hepatocyte iron transporter, by inducing its degradation. This leads to inhibition of iron absorption, iron release from hepatocytes, and further recycling by macrophage system. Synthesis of hepcidin is suppressed when increased amounts of iron are needed which is the case in IDA and hypoxia [9, 10].

Our aim was to determine serum hepcidin levels in patients with inflammatory bowel disease (IBD) as well to investigate whether there is a correlation of hepcidin levels with the disease activity in terms of whether hepcidin could be of use as a noninvasive marker of IBD activity.

## 2. Material and Methods

Complete medical records of 45 newly diagnosed IBD patients were evaluated. All patients were hospitalized in period between May 2014 and March 2015 at the Clinic of Gastroenterology and Hepatology, Clinical Center of Serbia. While hospitalized, enrolled patients underwent an ileocolonoscopy and diagnosis of IBD was established by current ECCO criteria [11]. Clinical scores used to evaluate the disease activity were Mayo Clinic score (remission ≤ 2 and active ≥ 3) and Crohn's disease activity index (CDAI) (remission < 150 and active ≥ 150) [12, 13]. As a control group, we analyzed the medical records of age- and sex-matched healthy volunteers, who had done their annual health checkup.

The exclusion criteria were as follows: (a) previous malignancy; (b) previous treatment with chemo- and/or radiotherapy; (c) evidence of other gastrointestinal, inflammatory, hematologic, hepatobiliary, kidney, pulmonary, and cardiovascular disease; (d) treatment with iron therapy and nonsteroidal anti-inflammatory drugs, as well as recent blood transfusions.

For all patients and healthy volunteers, complete blood counts (CBC) with automated differential counts were performed. Morning prior ileocolonoscopy, after an overnight fast, peripheral blood (2 ml) samples were collected from the cubital vein. CBC analysis was performed in the samples anticoagulated with EDTA within 4 hours after collection, using Coulter® LH750 Hematology Analyzer (Beckman Coulter, USA). Serum iron, TIBC, and UIBC as well as vitamin B12 and folic acid were assessed with an electrochemiluminesence immunoassay, and sTfR as well as CRP was assessed using an immunoturbidimetric method. Serum levels of hepcidin were determined with commercially available enzyme-linked immunosorbent assay (DRG Instruments, Marburg, Germany). During this study, laboratory was included in Beckman Coulter's Electronic Quality Assurance Program (Hematology Interlaboratory Quality Assurance Program).

The study was approved by the Institutional Ethical board (approval number 1447-7, Clinical Center of Serbia) and was performed in accordance with principles of Helsinki declaration. Informed written consent was obtained from all recruited subjects.

Statistical analysis was preformed using the SPSS ver. 20.0 (SPSS Inc., Chicago, IL, USA). Demographic characteristics, as well as clinical and histopathological characteristics were presented descriptively. Continuous variables were expressed as mean ± standard deviation (SD). Kolmogorov-Smirnov test was used for the estimation of normality distribution. Biochemical parameters were analyzed using Mann-Whitney test. The clinical and histopathological variables among groups were compared using Kruskal-Wallis test. Sensitivity, specificity, and the cut-off values were calculated according to the receiver-operator characteristic (ROC) analysis. Youden index was used for the calculation of the optimal cut-off values. Correlation was examined using Pearson's correlation test. A value of *P* < 0.05 was considered statistically significant.

## 3. Results

In our cohort, there were 23 patients (51%) with histologically proven UC, 22 patients (49%) with CD, and 45 age- and sex-matched healthy individuals. Regarding gender distribution in our cohort of patients, 27 (60%) were male, while 18 (40%) were female gender. Histopathological stage of the disease varied from 2.1, which meant that remissive disease until 5.2 is highly active disease, with median of 3.9, which is moderately active disease. Majority of patients had moderately active to highly active disease (75%), while 11 patients (25%) had remissive disease (Table 1).

**Figure 1:**
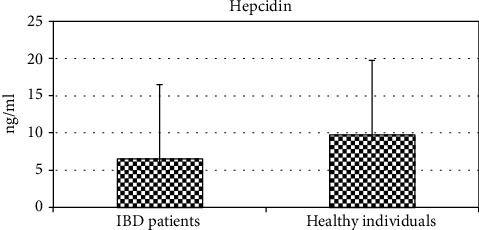
Hepcidin levels in IBD patients and healthy individuals.

There was a high statistically significant difference between IBD patients and controls in levels of hepcidin (*P* < 0.01) (Figure 1). Serum hepcidin levels were significantly higher in the control group (9.77 ± 2.71 vs. 6.40 ± 2.42 ng/ml, *P* = 0.001) (Table 2). However, when we analyzed hepcidin serum values between patients with CD and UC, we found no significant difference (6.59 ± 2.54 vs. 6.38 ± 2.67) (Table 3). Moreover, there was no statistically significant difference in hepcidin levels with regard to disease activity (*P* > 0.05). Namely, hepcidin values did not significantly differ in patients in remission when compared to patients with active disease (6.85 ± 2.34 vs. 6.26 ± 2.66). There was no statistically significant correlation of serum hepcidin with CRP, ESR, Mayo score, or CDAI, respectively (*P* > 0.05) (data not shown).

**Figure 2:**
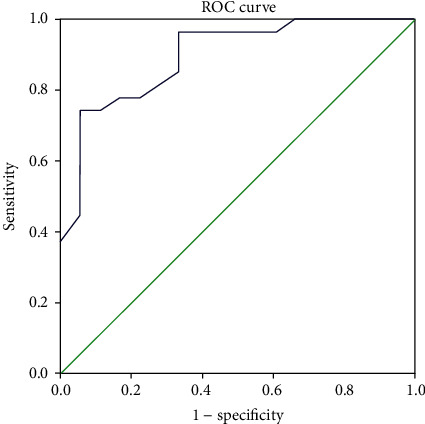
ROC curve analysis for hepcidin levels in IBD patients with anemia.

When establishing the diagnosis of IDA and ACD using iron, TIBC, ferritin, and transferrin, results showed that 18 (40%) patients had IDA and 27 had ACD (60%). Serum hepcidin levels were significantly different between these two groups, namely, patients with IDA had significantly lower levels of hepcidin when compared to patients with ACD (4.55 ± 1.67 vs. 8.22 ± 2.12, *P* < 0.01). ROC curve analysis for hepcidin in IBD patients with ACD (Figure 2) had the best cut-off value of 4.7 (AUC = 0.895, 95% CI 0.802-0.988, Se = 93% and Sp = 77%, PPV 85.7%, and NPV 80.6%).

Serum ferritin levels (394 ± 515 vs. 119 ± 124 ng/ml, *P* = 0.001) as well serum hepcidin to ferritin ratio (0.34 ± 0.17 vs. 0.22 ± 0.09, *P* = 0.02) were significantly higher in the control group when compared to IBD patients (Table 2). However, we did not find a statistically significant difference in values of both of these parameters with regard to CD or UC (*P* > 0.05). Additionally, there was no statistically significant difference in values of all examined parameters Er, Hgb, MCV, Hct, Tr, ESR, TIBC, UIBC, B12, folic acid, and fibrinogen between UC and CD patients (Table 3).

However, we found a statistically significant difference in values of transferrin saturation and sTfR (*P* < 0.01) in IBD patients when compared with the control group (Table 2). Additionally, there was a statistically significant negative correlation between values of iron, TIBC, and sTfR (*P* < 0.05). When we analyzed only IBD patients, we found no statistically significant correlation between values of sTfR and disease activity presented by Mayo score, CDAI, endoscopic, and histopathological findings (*P* < 0.01). However, we found a statistically significant negative correlation of sTfR and TIBC with hepcidin (*P* < 0.01).

## 4. Discussion

Hepcidin is considered to be a major regulatory factor of iron metabolism. In patients with IBD, inflammatory cytokines have direct influence on hepcidin synthesis and iron metabolism, leading to the development of ACD. Furthermore, influence of inflammation is even more pronounced in patients unresponsive to therapy and with more advanced disease [14]. Hepcidin levels in these patients are elevated, in contrary to patients with IDA, where hepcidin levels are low, so the absorption of iron and its releasement from the stores could be facilitated [14]. Recent studies have recognized hepcidin as a potential novel marker of differentiation of IDA from ACD in IBD patients [1]. Bearing in mind that patients with IBD could develop IDA or ACD, as well as IDA and ACD together, various studies have examined the relevance of hepcidin as a marker of inflammation [5, 8, 9, 12–14]. The results of previous investigations did not provide consistent results, which further highlights the need for investigation of hepcidin in inflammatory states such as IBD.

Results of the study of Arnold et al. [15] demonstrated lower serum hepcidin levels in IBD patients when compared with healthy controls. Namely, Arnold et al. demonstrated that IBD patients without anemia, as well as anemic IBD patients had significantly lower levels of hepcidin when compared to healthy individuals. These findings emphasize hepcidin significance in the initiation as well as in the maintenance of intestinal inflammation. Furthermore, investigation of Mecklenburg et al. [10] showed significantly reduced hepcidin levels in both anemic and nonanemic IBD patients when compared to healthy individuals, implying that iron levels regardless of inflammation regulate hepcidin synthesis. However, Oustamanolakis et al. [16] showed higher serum hepcidin levels in IBD patients, whereas Bergamaschi et al. [9] found no difference in serum hepcidin level between IBD patients and control group. These discrepancies in findings could exist due to heterogeneity of examined study groups, as well as the influence of various methods of serum hepcidin detection [9, 13].

In our cohort of patients, hepcidin levels were significantly lower than in control group. Having in mind that hepcidin is reliable marker of iron metabolism and that our cohort of patients is mostly consisted of newly diagnosed IBD patients with more pronounced IDA, our results are in concordance with Arnold et al. as well as Mecklenburg et al. [10, 15].

Furthermore, we have not found significant difference among Crohn's disease and ulcerative colitis group regarding the level of hepcidin, as well as in the group of active and remissive IBD patients. This result is in concordance with majority of previous investigations. Bergamaschi et al. and Paköz et al. as well as Arnold et al. have not found difference in hepcidin levels regarding different IBD types [9, 15, 17]. However, Oustamanolakis et al. [16] reported positive correlation between hepcidin level and disease activity in patients with ulcerative colitis. These differences may be explained by subjectivisms in the assessment of clinical activity, especially in CDAI score, as well as ethnic and geographic differences, having in mind that our cohort consisted of all Caucasian subjects, all hospital based, and presented as single-center experience.

Common for previous findings, as well as ours, is a positive correlation of hepcidin and ferritin levels, which leads to assumption that iron deficiency is more potent regulator of hepcidin level than inflammation. Our results demonstrate that in individuals with active IBD and associated IDA, hepcidin levels are low and well correlated with ferritin. Such is confirmed by Oustamanolakis et al. [16] as they found lower levels of hepcidin in IBD patients with significant IDA.

We mentioned earlier that Bergamaschi et al. [9] found no significant difference within levels of hepcidin in IBD and control group. This result is in concordance with the results of our investigation. Moreover, Bergamaschi et al. did not notice significant difference in between groups in parameters of iron status. Furthermore, results of their study suggested that hepcidin correlated negatively with sTfR-F index, which is completely in concordance to our results.

We did not establish any significant connection in examined laboratory markers (Er, Hgb, MCV, Hct, Plt, ESR, TIBC, UIBC, and fibrinogen) between UC and CD patients. Moreover, no correlation was found in parameters of iron status with inflammation CDAI and Mayo index, endoscopic, and histopathology. However, we found negative correlation of sTfR with hepcidin. These results could place sTfR as a possible indicator of iron requirement in patients with IBD and tailor adequate therapy modalities of anemia in IBD.

In conclusion, results of our study suggest that hepcidin is a potential marker for IDA in patients with IBD. Used in combination with other parameters of iron status could be used in everyday clinical practice to firmly establish the diagnosis of IDA in order to prevent further complications of IDA in IBD.

## Figures and Tables

**Table 1:** IBD patient's characteristics.

Age (mean ± SD)	47.42 ± 15.40
Gender (male/female, %)	27/18 (60/40)
UC (%)	23 (51)
CD (%)	22 (49)
Histopathological activity (mean)	3.9
Active disease (%)	34 (75)
Remissive disease (%)	11 (25%)

**Table 2:** Iron metabolism parameters in IBD patients and healthy subjects.

	IBD patients	Healthy individuals	*P*
Hepcidin (*η*g/ml)	6.40 ± 2.42	9.77 ± 2.71	0.001
Ferritin (*η*g/ml)	119 ± 124	394 ± 515	0.001
Hepcidin/ferritin ratio	0.22 ± 0.09	0.34 ± 0.17	0.02
Saturation of transferrin (%)	28.52 ± 20.85	34.84 ± 27.73	0.01

**Table 3:** Parameters of anemia in IBD patients.

	CD	UC	*P*
Hgb (g/L)	102.31	104.82	0.06
MCV (f/L)	77.3	77.72	0.97
ESR (mm/h)	37.69	37.01	0.95
CRP (g/L)	32.62	27.82	0.31
Fe (*μ*mol/L)	9.17	10.81	0.07
TIBC (*μ*mol/L)	62.86	59.24	0.14
UIBC (*μ*mol/L)	40.53	39.12	0.18
sTfR (mg/L)	2.5	3.3	0.33
Hepcidin (*η*g/ml)	6.59	6.38	0.07

## Data Availability

The data used to support the findings of this study are available from the corresponding author upon request.
